# Pre-existing maternal cardiovascular disease and the risk of offspring cardiovascular disease from infancy to early adulthood

**DOI:** 10.1093/eurheartj/ehae547

**Published:** 2024-09-04

**Authors:** Muhammad Zakir Hossin, Kalliopi Kazamia, Jonas Faxén, André Rudolph, Kari Johansson, Anna Sandström, Neda Razaz

**Affiliations:** Department of Medicine Solna, Clinical Epidemiology Division, Karolinska Institutet, Karolinska University Hospital, D1: 04, Stockholm SE-171 76, Sweden; School of Population and Public Health, University of British Columbia, 2206 East Mall, Vancouver BC V6T 1Z3, Canada; Department of Pediatric Cardiology, Stockholm-Uppsala, Karolinska University Hospital, Stockholm, Sweden; Department of Women's and Children's Health, Karolinska Institutet, Stockholm, Sweden; Department of Cardiology, Karolinska University Hospital, Stockholm, Sweden; Department of Physiology and Pharmacology, Karolinska Institutet, Stockholm, Sweden; Department of Pediatric Cardiology, Stockholm-Uppsala, Karolinska University Hospital, Stockholm, Sweden; Department of Medicine, Division of Rheumatology, Karolinska Institutet, Stockholm, Sweden; Department of Medicine Solna, Clinical Epidemiology Division, Karolinska Institutet, Karolinska University Hospital, D1: 04, Stockholm SE-171 76, Sweden; Division of Obstetrics, Department of Women’s Health, Karolinska University Hospital, Stockholm, Sweden; Department of Medicine Solna, Clinical Epidemiology Division, Karolinska Institutet, Karolinska University Hospital, D1: 04, Stockholm SE-171 76, Sweden; Division of Obstetrics, Department of Women’s Health, Karolinska University Hospital, Stockholm, Sweden; Department of Medicine Solna, Clinical Epidemiology Division, Karolinska Institutet, Karolinska University Hospital, D1: 04, Stockholm SE-171 76, Sweden

**Keywords:** Maternal cardiovascular disease, Paternal cardiovascular disease, Offspring cardiovascular disease, Pregnancy, Developmental programming, Negative-control exposure

## Abstract

**Background and Aims:**

A variety of maternal heart conditions are associated with abnormal placentation and reduced foetal growth. However, their impact on offspring’s long-term cardiovascular health is poorly studied. This study aims to investigate the association between intrauterine exposure to pre-existing maternal cardiovascular disease (CVD) and offspring CVD occurring from infancy to early adulthood, using paternal CVD as a negative control.

**Methods:**

This nationwide cohort study used register data of live singletons without major malformations or congenital heart disease born between 1992 and 2019 in Sweden. Hazard ratios (HRs) and 95% confidence intervals (CIs) were estimated using Cox proportional hazards models, adjusted for essential maternal characteristics. Paternal CVD served as a negative control for assessment of unmeasured genetic and environmental confounding.

**Results:**

Of the 2 597 786 offspring analysed (49.1% female), 26 471 (1.0%) were born to mothers with pre-existing CVD. During a median follow-up of 14 years (range 1–29 years), 17 382 offspring were diagnosed with CVD. Offspring of mothers with CVD had 2.09 times higher adjusted HR of CVD (95% CI 1.83, 2.39) compared with offspring of mothers without CVD. Compared with maternal CVD, paternal CVD showed an association of smaller magnitude (HR 1.49, 95% CI 1.32, 1.68). Increased hazards of offspring CVD were also found when stratifying maternal CVD into maternal arrhythmia (HR 2.94, 95% CI 2.41, 3.58), vascular (HR 1.59, 95% CI 1.21, 2.10), and structural heart diseases (HR 1.48, 95% CI 1.08, 2.02).

**Conclusions:**

Maternal CVD was associated with an increased risk of CVD in offspring during childhood and young adulthood. Paternal comparison suggests that genetic or shared familial factors may not fully explain this association.

## Structured Graphical Abstract

**Structured Graphical Abstract:**
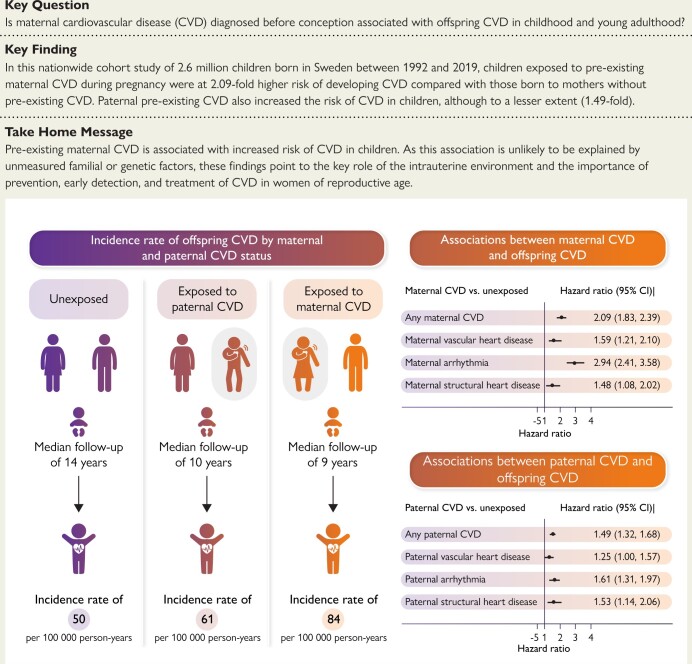
Plots showing incidence rates and adjusted hazard ratios of offspring cardiovascular disease according to the presence of pre-existing maternal and paternal cardiovascular disease: singleton offspring live born without major malformations in Sweden 1992–2019. CI, confidence interval; CVD, cardiovascular disease.

## Introduction

Maternal cardiovascular disease (CVD) is a major cause of pregnancy complications, infant morbidity and maternal mortality, with increasing prevalence worldwide.^1,2^ Pregnancy-related haemodynamic changes, which are crucial for foetal growth, may compromise cardiovascular adaptability in women with pre-existing CVD, impacting the intrauterine environment and offspring health.^3,4^ The foetal/developmental programming hypothesis suggests that exposure to suboptimal intrauterine environment can influence the risk of cardiovascular and other chronic diseases later in life.^5–7^ Observational epidemiological studies have shown that maternal heart diseases in pregnancy, both congenital and acquired, are associated with increased risks of adverse perinatal outcomes (e.g. preterm birth and reduced foetal growth),^8–10^ which in turn may contribute to the programming of future health and disease of offspring.^4,11^ However, evidence on the long-term programming effect of maternal CVD on offspring health including CVD is currently scarce.^4,10^

**Figure 1:**
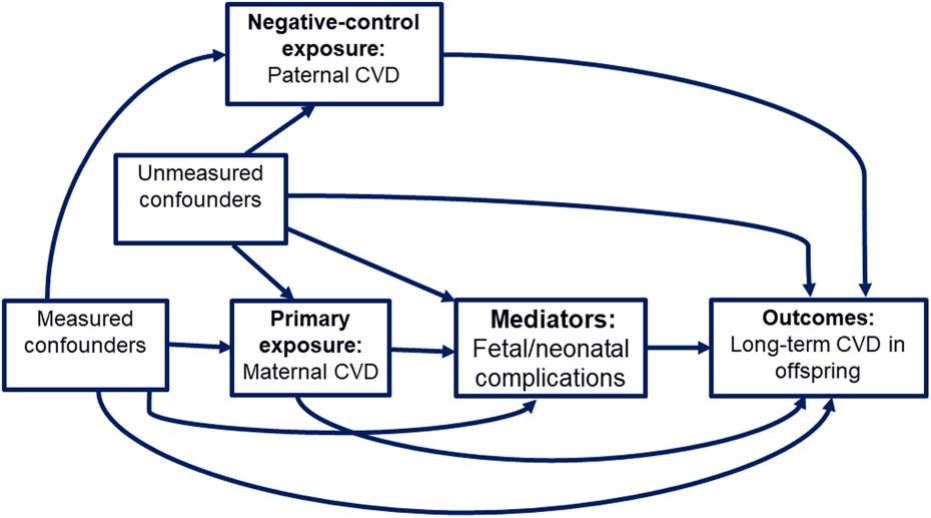
Illustration of the causal structure of the study, including the negative control framework. Measured confounders include maternal socio-demographic and clinical characteristics, while unmeasured confounders refer to shared genetics and family environment. Potential mediators of interest are preterm birth and small-for-gestational age. Outcomes are children’s cardiovascular diseases diagnosed up to age 29 years

A methodological challenge in examining the role of the maternal intrauterine environment is the presence of unmeasured common genetic variants that may influence both maternal and child CVD. In this context, a negative-control exposure design that compares the maternal–offspring association with the paternal–offspring association may provide important insights into the underlying biological vs. confounding mechanisms.^12,13^ Given that paternal CVD shares the same sources of unmeasured confounding as maternal CVD and does not directly influence the intrauterine environment (*Figure 1*), paternal CVD as a negative-control exposure can exploit the similarity of confounding structure and thereby serve as an innovative tool to evaluate shared genetic and environmental confounding.

In this large nationwide cohort study in Sweden, we aimed to evaluate the association between pre-existing maternal CVD in pregnancy and offspring CVD during childhood and young adulthood, using paternal CVD as a negative control. We hypothesized that the intrauterine environment plays a key role in transmitting CVD risk to the offspring. Consequently, we anticipated a stronger maternal–offspring than paternal–offspring association of CVD, with the former reflecting both intrauterine and genetic influences, while the latter ruling out the influence of the *in utero* environment.

## Methods

### Data sources

Data for this nationwide cohort study were primarily retrieved from the Swedish Medical Birth Register that covers ≥98% of all births in Sweden since 1973 and contains information on maternal and perinatal characteristics including pregnancy and neonatal complications.^14^ The medical birth register was cross-linked to other national registers including the National Patient Register (inpatient and outpatient specialist care),^15,16^ the Cause of Death Register,^17^ the Total Population Register,^18^ and the Education Register^19^ for identification of the exposure, outcomes, and background characteristics (see Supplementary data online, *Methods* for details of the registers). All data were pseudonymized and linked through an encrypted unique identifier. The study was approved by the Swedish Ethical Review Authority.

### Study population

**Figure 2:**
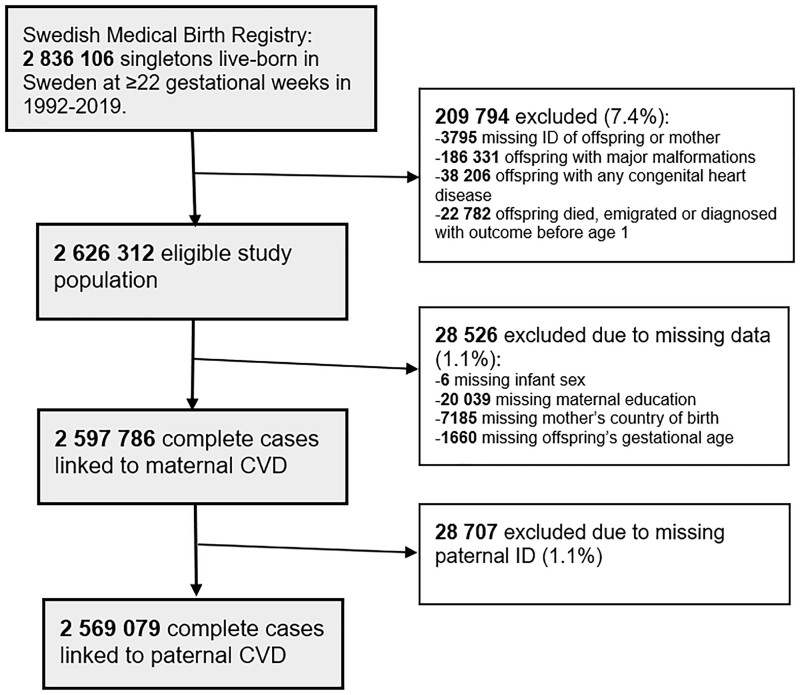
Flow diagram of the study samples.

Between 1 January 1992 and 31 December 2019, the Medical Birth Register recorded information for 2 836 106 singletons live born at ≥22 completed gestational weeks of primiparous or parous mothers (*Figure 2*). We excluded (i) births where either the mothers or the children had missing national registration number; (ii) children diagnosed with any major malformations or congenital heart diseases in the first year of life or during follow-up [see International Classification of Disease (ICD) codes in Supplementary data online, *Table S1*]; and (iii) children who died (*n* = 7436), emigrated (*n* = 7951), or had CVD (*n* = 7395) within the first year of life. Among 7436 infants who died before 1 year of age, 115 (1.5%) were pre-natally exposed to maternal CVD and 1052 (14.2%) had a CVD diagnosis. Among those who met the eligibility criteria (*n* = 2 626 312), a total of 2 597 786 (98.9%) children with complete data on the study variables were included in the statistical analyses. Complete paternal information was available for 2 569 079 (98.9%) of these children (*Figure 2*).

### Measures

#### Exposures

The primary exposure was pre-existing maternal CVD defined as any primary or secondary diagnosis of CVD identified any time before the date of conception [date of birth—(gestational age + 14 days)], using the ICD-9 or ICD-10 codes (see Supplementary data online, *Table S2*). A cardiovascular diagnosis was identified based on at least one ICD record in the inpatient register (since 1987) or at least two ICD records in the outpatient specialist care register (since 2001). Although our main exposure includes maternal CVD of any type, three major subgroups of maternal CVD were also examined separately: (i) vascular disease (ischaemic heart disease, cerebrovascular, and hypertensive diseases); (ii) arrhythmia; and (iii) structural heart disease (cardiomyopathy, heart failure, valvular, and congenital heart diseases). We opted to collapse specific maternal CVD conditions into broad subgroups considering the lack of adequate statistical power to stratify CVD into finer categories. Pre-existing paternal CVD and subgroups were assessed using the same definitions as maternal CVD.

#### Outcomes

The outcome of interest was the first offspring CVD event recorded after the first year of life. The primary outcome was CVD of any type (ICD-9: 390–459; ICD-10: I00–I99). Outcome information was defined as diagnosis of or death due to CVD based on ≥1 ICD record in the inpatient or the Cause of Death register, or ≥2 ICD records in the outpatient specialist care register (see ICD codes in Supplementary data online, *Table S1*). In addition, two relatively common subgroups of offspring CVD were analysed as secondary outcomes: (i) arrhythmia and (ii) vascular diseases (i.e. hypertensive disease, cerebrovascular disease, and ischaemic heart disease). To avoid measuring CVD as a neonatal complication (e.g. neonatal stroke, birth defect, and extreme preterm birth), all children recorded with an ICD code for CVD before the first year of life were excluded. Follow-up started from the first birthday and continued until the date of outcome diagnosis, emigration, death, or 31 December 2020, whichever occurred first. A total of 4235 (.2%) offspring were censored due to deaths and 87 401 (3.4%) due to emigration. When analysing arrhythmia as a separate outcome, follow-up was started from birth given that some paediatric supraventricular arrhythmias (e.g. atrial flutter) are specifically incident among neonates and infants.

#### Covariates

The covariates selected *a priori* in the study include infant sex (male/female), birth year (5 year intervals), and mother’s age at delivery, parity, country of birth, cohabitation status, highest educational level, body mass index (BMI) in early pregnancy, smoking in early pregnancy, pre-gestational diabetes, chronic kidney disease, and history of any psychiatric disorders. Data on maternal disease covariates (see Supplementary data online, *Table S2*) were obtained from the National Patient Register or the Medical Birth Register, while data on maternal education and country of birth were retrieved from the Education Register and the Total Population Register, respectively. The paternal covariates available in the study were paternal age at the time of child’s birth, psychiatric disorders, and chronic kidney disease. Maternal and paternal psychiatric disorders were defined on the basis of at least one inpatient or outpatient visit listed with an ICD code (ICD-9: 290–319; ICD-10: F00–F99) any time before the date of conception.

### Statistical analyses

All statistical analyses were performed using Stata (version 17.0). The cumulative incidence of offspring CVD, comparing children exposed to pre-existing maternal CVD vs. those unexposed, was illustrated using cumulative hazard plots. The crude incidence rates among both exposed and unexposed groups were calculated in the unit of 100 000 person-years, and the rate differences were reported with 95% confidence intervals (CIs). Cox proportional hazard regression models were fitted, with offspring age as the underlying time scale, to estimate the hazard ratios (HRs) for the associations between pre-existing maternal CVD and risk of CVD in the offspring. The proportional hazards assumption was assessed for the exposure, using both statistical test and visual inspection through log-minus-log survival plot (see Supplementary data online, *Figure S1*), which did not suggest strong evidence of violation of the assumption. Robust standard errors were estimated to account for intra-cluster correlations between siblings born to the same mother. The associations were adjusted for offspring’s sex and birth year and mother’s age at delivery, parity, educational level, country of birth, pre-gestational diabetes, chronic kidney disease, history of any psychiatric disorders as well as paternal CVD. We also calculated attributable fraction in the exposed (AFE) to evaluate the excess burden of CVD among the exposed children that could be potentially attributed to maternal CVD, using the following formula^20^: (HR − 1)/HR. Assuming a causal effect of the exposure on the outcome, the AFE measure quantifies the proportion of cases among the exposed population that would not have occurred had the exposure been absent.^20,21^

As no evidence of effect modification by offspring sex was found, the analysis was conducted on both males and females combined. However, considering the differences in CVD composition between older and younger parents, we explored potential effect modifications in the associations by parental age at childbirth. We further investigated whether the associations were modified by parent’s psychiatric disorders given the very high co-occurrence of such disorders with CVD,^22^ both of which may interact to increase CVD risk in offspring.

#### Negative-control exposure analysis

To assess potential confounding by shared familial factors (e.g. genetic factors) in the association between maternal and offspring CVD, we conducted a negative-control exposure analysis using paternal CVD. A negative-control exposure ideally shares the same unmeasured confounders as the primary exposure and is not associated with the outcome through the hypothesized causal pathway (*Figure 1*).^12,23^ Given the absence of the intrauterine environment in fathers, we assumed a weaker association between paternal and offspring CVD. While a null association would imply no evidence of unmeasured confounding, any observed paternal–offspring association would indicate the presence of such confounding in the maternal–offspring association. The paternal models we fitted were analogous to the maternal models, with additional adjustment for maternal CVD and paternal age at childbirth. Adjustment for maternal CVD and risk factors were made considering their possible correlations with paternal CVD through assortative mating.^24,25^ The difference between the maternal–offspring and paternal–offspring CVD associations was quantified and statistically tested using the Wald test.

#### Causal mediation analysis

We considered preterm birth (i.e. delivery before the 37th week of completed gestation) and small-for-gestational age (SGA) as potential mediators since they were previously found to be influenced by maternal heart disease^8,26^ and are risk factors for CVD in later life.^27,28^ SGA was defined as birthweight-for-gestational age of <10th percentile according to the sex-specific Swedish reference curve for normal foetal growth.^29^ The mediating impact of preterm birth in the estimated association between maternal and offspring CVD was examined by decomposing the total exposure effect into natural indirect effect that may pass through preterm birth and the natural direct effect involving other mechanisms. The generalized Poisson linear model was used for the purpose of the mediation analysis, which was conducted under the assumptions that there were no unmeasured confounders affecting the exposure–outcome, exposure–mediator, and mediator–outcome associations.^30^

#### Sensitivity analyses

Several sensitivity analyses were carried out to evaluate the robustness of the main results. First, due to the relatively high proportion of missing values on maternal BMI (10.9%), smoking (3.9%), and cohabitation status (5.5%), adjustment for these risk factors was made in a multiple imputation analysis, using 15 imputed datasets. Second, while maternal/paternal CVD was stratified into three broad groups considering the statistical power of the associations under investigation, additional analysis was conducted by stratifying maternal CVD into seven categories (i.e. ischaemic heart disease, cerebrovascular disease, hypertensive disease, arrhythmia, cardiomyopathy/heart failure, valvular heart disease, and congenital heart disease). Third, to examine whether the timing of CVD diagnosis in mothers influences the risk of offspring CVD, we stratified maternal CVD diagnoses into three categories of duration: < 2, 2–5, and >5 years before conception. Fourth, we tested the robustness of the maternal–offspring CVD associations by excluding mothers diagnosed with pre-eclampsia and gestational hypertension during the index pregnancy. Fifth, the analysis was restricted to children born from 2001 onward when the outpatient register was established in Sweden. Sixth, to partly complement the outpatient register before the year 2001, we extracted additional maternal CVD diagnoses (*n* = 494) from the Medical Birth Register in 1992–2001. Finally, since CVD diagnoses in mothers not born in Sweden are less likely to be captured, sensitivity analysis was undertaken on mothers born in Sweden only.

## Results

Of the 2 597 786 offspring included in the final statistical analyses, we identified 26 471 (1.0%) offspring born to mothers with any pre-existing CVD. Among mothers with CVD, the most common subtype was arrhythmia (35%), followed by hypertensive disease (17%), cerebrovascular disease (10%), and valvular heart disease (7%), as shown in Supplementary data online, *Table S3*. We also identified 5171 children born to mothers with any congenital heart disease. While vascular heart diseases were more prevalent in older mothers (>34 years, Supplementary data online, *Table S3*), there were relatively higher percentages of arrhythmia and congenital heart disease in younger mothers (<25 years).

Mothers with pre-existing CVD were more likely to be older (>34 years), multiparous (parity of ≥4), and born in Sweden and have a higher prevalence of obesity (BMI ≥30 kg/m^2^), psychiatric disorders, pre-gestational diabetes, and chronic kidney disease (*Table 1*). The proportion of preterm birth (<37 weeks) was higher in offspring of mothers with CVD, compared with mothers without CVD.

**Table 1:** Maternal and offspring characteristics according to maternal pre-existing cardiovascular disease status: singleton offspring live-born without major malformations in Sweden 1992–2019

	Total population^a^ (*n* = 2 626 312)	Pre-existing maternal CVD	
		No (*n* = 2 599 724)	Yes (*n* = 26 588)
Offspring characteristics % (*n*)			
Sex			
Male	50.9 (1 336 145)	50.9 (1 322 671)	50.7 (13 474)
Female	49.1 (1 290 161)	49.1 (1 277 047)	49.3 (13 114)
Missing	0.0 (6)	0.0 (6)	0.0 (0)
Birth year			
1992–94	12.1 (316 814)	12.2 (315 867)	3.6 (947)
1995–99	15.6 (408 837)	15.6 (406 750)	7.8 (2087)
2000–04	15.8 (414 523)	15.8 (411 270)	12.2 (3253)
2005–09	17.7 (465 688)	17.7 (460 611)	19.1 (5077)
2010–14	19.1 (501 274)	19.0 (494 345)	26.1 (6929)
2015–19	19.8 (519 176)	19.7 (510 881)	31.2 (8295)
Preterm birth			
No	95.5 (2 509 598)	95.6 (2 485 194)	91.8 (24 404)
Yes	4.4 (115 054)	4.3 (112 870)	8.2 (2184)
Missing	.1 (1660)	.1 (1660)	0.0 (0)
Small-for-gestational age			
No	93.5 (2 455 764)	93.5 (2 431 323)	91.9 (24 441)
Yes	6.2 (162 504)	6.2 (160 426)	7.8 (2078)
Missing	.3 (8044)	.3 (7975)	.3 (69)
Maternal characteristics %(*n*)			
Age at delivery (years)			
≤ 19	1.6 (42 205)	1.6 (42 033)	.6 (172)
20–24	13.9 (365 457)	14.0 (363 076)	9.0 (2381)
25–29	32.5 (854 706)	32.6 (847 463)	27.2 (7243)
30–34	33.1 (868 880)	33.1 (859 758)	34.3 (9122)
≥ 35	18.9 (495 064)	18.7 (487 394)	28.8 (7670)
Parity			
1	42.9 (1 127 858)	43.0 (1 118 057)	36.9 (9801)
2	37.2 (976 505)	37.2 (966 164)	38.9 (10 341)
3	13.8 (363 533)	13.8 (359 253)	16.1 (4280)
≥ 4	6.0 (158 416)	6.0 (156 250)	8.1 (2166)
Country of birth			
Sweden	78.5 (2 062 536)	78.5 (2 039 541)	86.5 (22 995)
Other Nordic country	1.9 (49 814)	1.9 (49 421)	1.5 (393)
Non-Nordic country	19.3 (506 777)	19.4 (503 604)	11.9 (3173)
Missing	.3 (7185)	.3 (7158)	.1 (27)
Level of education (years)			
≤ 9	8.5 (224 208)	8.5 (221 956)	8.5 (2252)
10–11	15.4 (404 439)	15.4 (400 976)	13.0 (3463)
12	24.4 (641 956)	24.4 (635 055)	26.0 (6901)
13–14	14.9 (391 333)	14.9 (387 750)	13.5 (3583)
≥ 15	36.0 (944 337)	35.9 (934 038)	38.7 (10 299)
Missing	.8 (20 039)	.8 (19 949)	.3 (90)
Cohabitation with partner			
No	5.5 (144 312)	5.5 (142 791)	5.7 (1521)
Yes	89.0 (2 338 430)	89.0 (2 314 884)	88.6 (23 546)
Missing	5.5 (143 570)	5.5 (142 049)	5.7 (1521)
Smoking in early pregnancy			
No	86.3 (2 265 436)	86.3 (2 242 474)	86.4 (22 962)
Yes	9.9 (259 194)	9.9 (256 701)	9.4 (2 493)
Missing	3.9 (101 682)	3.9 (100 549)	4.3 (1133)
BMI in early pregnancy (kg/m^2^)			
Underweight (<18.5)	2.3 (59 334)	2.3 (58 790)	2.0 (544)
Normal weight (18.5–25)	54.6 (1 435 268)	54.7 (1 422 017)	49.8 (13 251)
Overweight (25–30)	22.2 (581 757)	22.1 (575 393)	23.9 (6 364)
Obesity (≥30)	10.1 (264 164)	10.0 (260 234)	14.8 (3 930)
Missing	10.9 (285 789)	10.9 (283 290)	9.4 (2499)
Any psychiatric disorders			
No	92.4 (2 426 298)	92.5 (2 404 600)	81.6 (21 698)
Yes	7.6 (200 014)	7.5 (195 124)	18.4 (4890)
Chronic kidney disease			
No	100.0 (2 625 580)	100.0 (2 599 274)	98.9 (26 306)
Yes	0.0 (732)	0.0 (450)	1.1 (282)
Pre-gestational diabetes			
No	99.6 (2 614 599)	99.6 (2 588 546)	98.0 (26 053)
Yes	.4 (11 713)	.4 (11 178)	2.0 (535)

BMI, body mass index; CVD, cardiovascular disease.

^a^The total population in this table refers to the eligible study population without exclusion of missing data.

**Figure 3:**
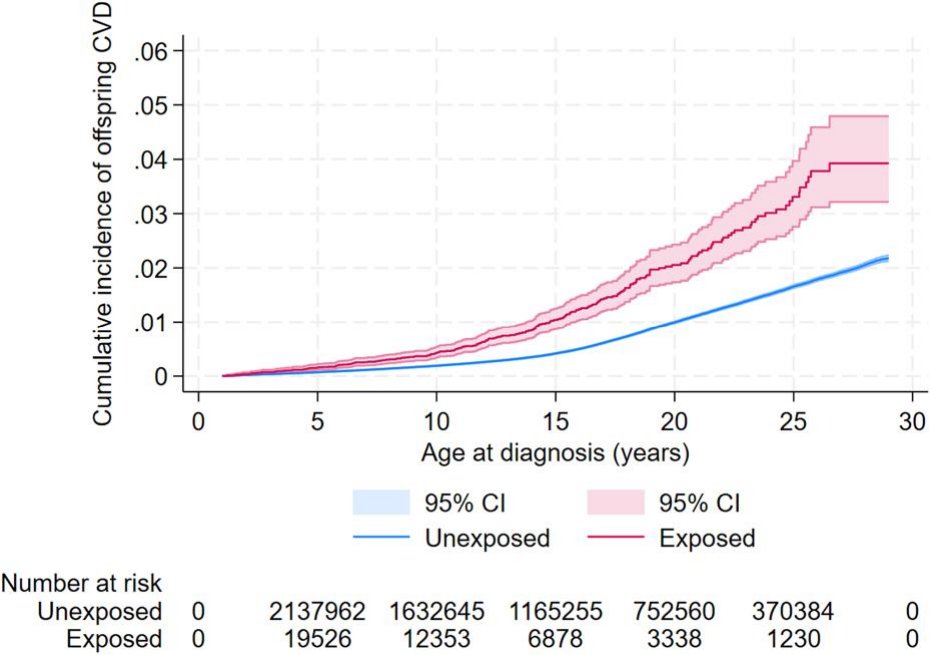
Unadjusted cumulative incidence of cardiovascular disease among offspring exposed and unexposed to pre-existing maternal cardiovascular disease.

During a median follow-up of 14 years (range 1–29 years), 17 382 (.7%) offspring developed CVD, with an overall incidence rate of 50 per 100 000 person-years. The offspring exposed to maternal CVD had 34.4 (95% CI 23.1, 45.7) additional cases of CVD per 100 000 person-years compared with those born to mothers without CVD (*Table 2*). Unadjusted cumulative incidence curves showed a higher cumulative hazard of CVD among offspring exposed to maternal CVD compared with the non-exposed (*Figure 3*).

**Table 2:** Unadjusted incidence rate, rate differences, and hazard ratios of offspring cardiovascular disease according to the presence of pre-existing maternal cardiovascular disease: singletons live-born without major malformations in Sweden 1992–2019 (*n* = 2 597 786)

Pre-existing maternal CVD	Outcome: any CVD in offspring				
	No. of events	Rate per 100 000 py^a^	Rate difference (95% CI)^a^	Unadjusted HR (95% CI)	AFE % (95% CI)^b^
Any CVD					
No	17 165	50.2	0.0 (Ref.)	1.00 (Ref.)	
Yes	217	84.6	34.4 (23.1, 45.7)	2.15 (1.88, 2.45)	52.2 (46.1, 58.4)
Pre-existing maternal CVD subgroups					
Vascular heart disease					
No	17 331	50.4	0.0 (Ref.)	1.00 (Ref.)	
Yes	51	69.4	19 (−.1, 38.0)	1.75 (1.33, 2.30)	37.1 (21.5, 52.2)
Arrhythmia					
No	17 280	50.3	0.0 (Ref.)	1.00 (Ref.)	
Yes	102	117.9	67.6 (44.7, 90.5)	3.14 (2.58, 3.82)	66.0 (59.9, 72.1)
Structural heart disease					
No	17 342	50.4	0.0 (Ref.)	1.00 (Ref.)	
Yes	40	69.3	18.9 (−2.6, 40.4)	1.86 (1.36, 2.54)	32.4 (14.8, 50.0)
	Outcome: Arrhythmia in offspring^c^				
Pre-existing maternal CVD					
Any CVD					
No	8292	22.3	0.0 (Ref.)	1.00 (Ref.)	
Yes	199	69.3	47.0 (37.3, 56.7)	3.47 (3.01, 4.00)	70.0 (66.2, 73.9)
Pre-existing maternal CVD subgroups					
Vascular heart disease					
No	8405	22.7	0.0 (Ref.)	1.00 (Ref.)	
Yes	24	29.5	6.8 (−5.0, 18.6)	1.45 (.97, 2.17)	14.8 (−29.1, 58.7)
Arrhythmia					
No	8289	22.4	0.0 (Ref.)	1.00 (Ref.)	
Yes	140	145.7	123.3 (99.1, 147.4)	7.43 (6.29, 8.79)	85.2 (82.8, 87.5)
Structural heart disease					
No	8394	22.7	0.0 (Ref.)	1.00 (Ref.)	
Yes	35	54.1	31.4 (13.5, 49.3)	2.71 (1.94, 3.77)	39.0 (16.1, 62.1)
	Outcome: Vascular heart disease in offspring				
Pre-existing maternal CVD					
Any CVD					
No	3591	10.4	0.0 (Ref.)	1.00 (Ref.)	
Yes	38	14.7	4.3 (−.4, 9.0)	1.75 (1.27, 2.41)	38.6 (20.5, 56.7)
Pre-existing maternal CVD subgroups					
Vascular heart disease					
No	3614	10.5	0.0 (Ref.)	1.00 (Ref.)	
Yes	15	20.4	9.9 (−.4, 20.2)	2.41 (1.45, 4.00)	53.9 (27.2, 81.0)
Arrhythmia					
No	3619	10.5	0.0 (Ref.)	1.00 (Ref.)	
Yes	10	11.5	1.0 (−6.2, 8.1)	1.42 (.76, 2.63)	22.5 (−30.8, 76.0)
Structural heart disease					
No	3623	10.3	0.0 (Ref.)	1.00 (Ref.)	
Yes	6	11.5	.2 (−8.5, 8.1)	1.28 (.58, 2.86)	3.8 (−1.13, 1.20)

AFE, attributable fraction in the exposed; CI, confidence interval; CVD, cardiovascular disease; HR, hazard ratio.

^a^The crude incidence rates and rate differences were calculated per 100 000 person-years.

**Figure 4:**
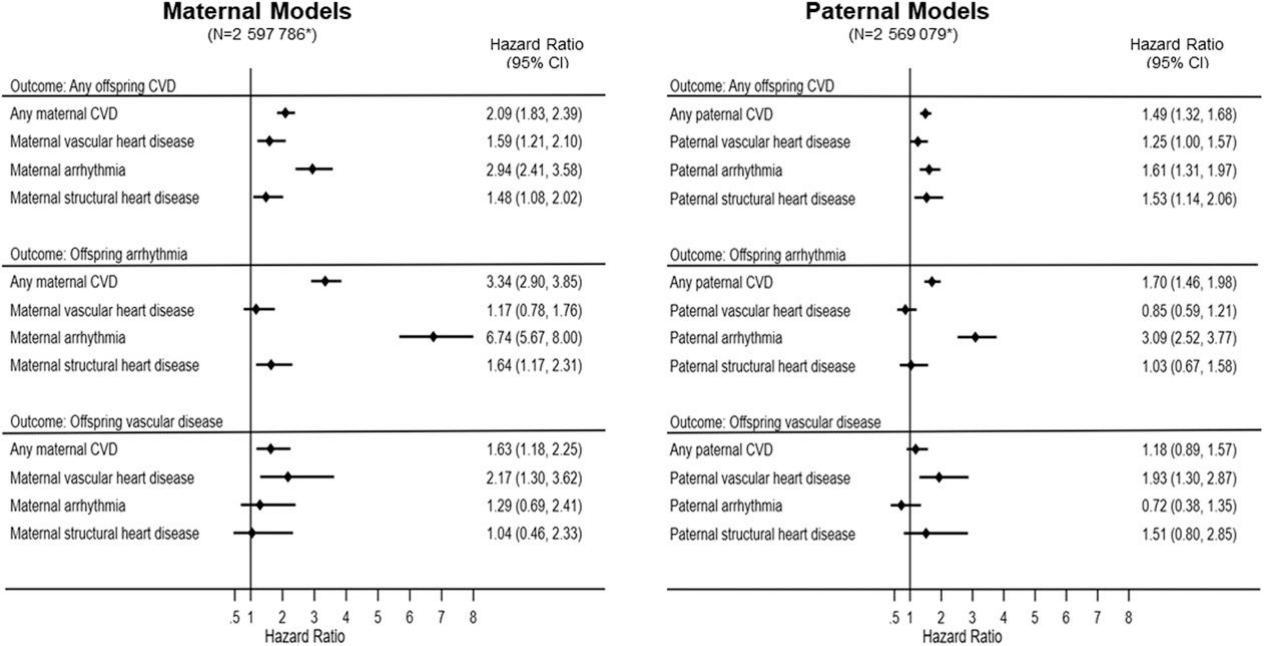
Plots showing adjusted hazard ratios of offspring cardiovascular disease outcomes according to the presence of pre-existing maternal and paternal cardiovascular disease: singleton offspring live-born without major malformations in Sweden 1992–2019. *Follow-up for offspring arrhythmia started from birth, instead of from first year of life (maternal model: *n* = 2 611 790; paternal model: *n* = 2 581 603). The hazard ratios were adjusted for offspring sex and birth year and mother’s age at delivery, parity, country of birth, level of education, pre-gestational diabetes, parents’ chronic kidney disease, and parental history of any psychiatric disorders. The maternal and paternal cardiovascular disease was mutually adjusted for each other. The paternal models were additionally adjusted for paternal age at childbirth. The subgroups of maternal/paternal cardiovascular disease were mutually adjusted for each other

^b^AFE indicates the percentage of the outcome in the exposed children that can be attributed to the exposure and was obtained from the adjusted HR shown in *Figure 4*. The 95% CI was obtained by bootstrapping, with 500 replications.

^c^Follow-up for offspring arrhythmia started from birth, instead of from first year of life (*n* = 2 611 790).

The adjusted Cox model suggested that exposure to maternal CVD was associated with 2.09-times higher hazard of CVD (adjusted HR 2.09, 95% CI 1.83, 2.39) compared with no exposure to maternal CVD (*Figure 4*). The AFE for this association was estimated as 52.2% (95% CI 46.1, 58.4; *Table 2*). Stratification by maternal CVD subgroups showed higher hazards of CVD in offspring exposed to maternal arrhythmia, vascular disease, and structural heart disease when compared with offspring of mothers without these CVD conditions (*Figure 4*).

Among the offspring with CVD, the majority of CVD cases were due to arrhythmia (38%), followed by hypertensive disease (12%) and cerebrovascular disease (8%) (see Supplementary data online, *Table S4*). Maternal CVD was associated with higher hazards of offspring arrhythmia (adjusted HR 3.34, 95% CI 2.90, 3.85) and vascular disease (adjusted HR 1.63, 95% CI 1.18, 2.25), compared with no maternal CVD. The hazard of arrhythmia was 6.74 times higher (95% CI 5.67, 8.00) in offspring born to mothers with arrhythmia compared with offspring of mothers without arrhythmia. Similarly, an increased hazard of vascular disease was observed in offspring exposed to maternal vascular disease (*Figure 4*).

The incidence rate differences and HRs of offspring CVD by pre-existing paternal CVD as a negative control are shown in Supplementary data online, *Table S5*. There were 10.8 (95% CI 3.6, 18.2) additional cases of CVD per 100 000 person-years in offspring with paternal CVD vs. no paternal CVD. Paternal CVD was associated with 49% higher hazard for offspring CVD (adjusted HR 1.49; 95% CI 1.32–1.68), although the magnitude of this association was smaller compared with that of maternal CVD (*Figure 4*). The formal statistical test suggests that the maternal–offspring association was 1.40 times larger than the paternal–offspring association (ratio of the two adjusted HRs 1.40; 95% CI 1.18, 1.68).

Increased hazards of CVD were also found in offspring exposed to paternal CVD subgroups, namely paternal vascular disease, arrhythmia, and structural heart disease. Paternal CVD showed 1.70-fold increased hazard for offspring arrhythmia (95% CI 1.46, 1.98) and no increased hazard for offspring vascular disease. Further, offspring born to fathers with arrhythmia or vascular disease showed 3.09-fold and 1.95-fold elevated hazards of arrhythmia and vascular disease, respectively. In general, the HR estimates of maternal CVD and subgroups (except for structural heart disease) tended to be larger than the respective HRs of paternal CVD and subgroups (*Figure 4*).

**Figure 5:**
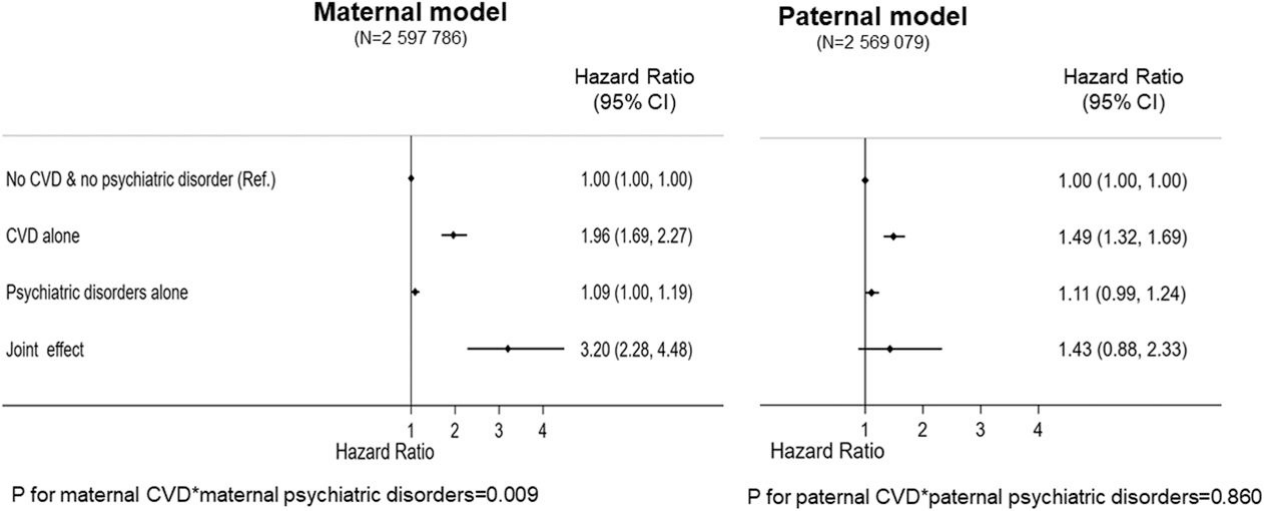
Main and joint effects of maternal cardiovascular disease and psychiatric disorders on offspring cardiovascular disease, using paternal cardiovascular disease and psychiatric disorders as negative controls: singleton offspring live-born without major malformations in Sweden 1992–2019. The hazard ratios were adjusted for offspring sex and birth year and mother’s age at delivery, parity, country of birth, level of education, pre-gestational diabetes, and parents’ chronic kidney disease. In addition, the maternal model was adjusted for paternal cardiovascular disease and psychiatric disorders, whereas the paternal model was adjusted for maternal cardiovascular disease and psychiatric disorders

A total of 598 events of CVD were recorded in offspring born to mothers with psychiatric disorders (*n* = 198 592). Of the 4832 offspring who were exposed to both maternal CVD and psychiatric disorders, 38 events of CVD were recorded (incidence rate 109 per 100 000 person-years). Maternal CVD combined with any comorbid psychiatric disorders was more strongly associated with offspring CVD (adjusted HR 3.20, 95% CI 2.28, 4.48) compared with maternal CVD with no psychiatric disorders (adjusted HR 1.96, 95% CI 1.69, 2.27; *Figure 5*). Maternal psychiatric disorders alone were associated with 9% increased hazard of CVD in offspring (adjusted HR 1.09, 95% CI 1.00, 1.19). No interaction was found between paternal CVD and psychiatric disorders (*Figure 5*). The association between maternal and offspring CVD did not vary across maternal age groups (see Supplementary data online, *Figure S2*).

SGA did not show a strong association with maternal CVD in the initial investigation and was dropped from the final mediation analysis. The causal mediation analyses revealed that preterm birth mediated only 5% of the association between maternal and offspring CVD (see Supplementary data online, *Table S6* and Supplementary data online, *Table S7*).

### Sensitivity analyses

Among the specific maternal CVD categories studied in sensitivity analysis, maternal ischaemic heart disease, hypertensive disease, arrhythmia, cardiomyopathy/heart failure, and valvular heart disease were associated with increased hazard of offspring CVD (see Supplementary data online, *Table S8*). The strength of the association between maternal and offspring CVD remained unattenuated after additional adjustments for maternal BMI, smoking, and cohabitation status in the multiple imputation analysis (see Supplementary data online, *Table S9*). The HRs were slightly elevated when mothers with pre-eclampsia and gestational hypertension were excluded (see Supplementary data online, *Table S10*). Maternal CVD diagnosed within 2 years preceding conception showed an HR of 2.46 on offspring CVD (95% CI 2.00, 3.01) in contrast to an HR of 1.93 (95% CI 1.51, 2.48) when maternal CVD was diagnosed at least 5 years before conception (see Supplementary data online, *Table S11*). However, the overlapping 95% CIs for the two HRs indicate no significant difference. The analyses restricted to later-born cohorts i.e. offspring born from 2001 onward (see Supplementary data online, *Table S12*) or to children of Swedish-born mothers (see Supplementary data online, *Table S13*) yielded results consistent with those obtained in the main analyses. The maternal–offspring CVD association was robust when CVD diagnoses from the medical birth register were additionally included in the definition of pre-existing maternal CVD (see Supplementary data online, *Table S14*).

## Discussion

### Main findings

In this nationwide cohort study of 2.6 million children, we found that individuals exposed to pre-existing maternal CVD *in utero* had a higher risk of acquiring CVD in childhood and early adulthood. In particular, risks of arrhythmia and vascular disease were increased. Maternal arrhythmia and vascular and structural heart diseases were associated with an elevated risk of CVD in offspring. Paternal CVD was also associated with an increased risk of offspring CVD. However, this association was weaker in magnitude compared with the association found with maternal CVD (*Structured Graphical Abstract*). Furthermore, among offspring born to mothers with pre-existing CVD, the presence of psychiatric comorbidity further heightened the risk of CVD.

### Comparison with existing literature

To our knowledge, no previous study has systematically investigated the influence of the intrauterine environment underlying the associations between maternal cardiovascular conditions and offspring CVD risk. Existing studies often have limited sample size, lack data on specific maternal or child cardiovascular conditions, and predominantly focus on traditional risk factors for CVD, such as BMI, blood pressure, total cholesterol level, or diet.^31,32^ Our findings are aligned with the broader literature suggesting that children exposed to adverse maternal cardiovascular risk profile during pregnancy may have elevated cardiovascular health risks.^32–34^

The previous literature examining the differential transmission of CVD or its risk factors along maternal and paternal lines yielded inconsistent results.^35^ In line with our study, some studies showed a stronger maternal–offspring association,^34,36^ while some found fathers as important as mothers for intergenerational transmission of CVD risk.^33,37^ Data from the Framingham Heart Study showed that maternal cardiovascular health was a more robust predictor of CVD-free survival among offspring compared with paternal cardiovascular health.^34^ In contrast, a Norwegian cohort study found that maternal–offspring associations for various cardiovascular risk factors were largely similar to paternal–offspring associations,^33^ arguing against maternal effects through intrauterine mechanisms. However, the maternal cardiovascular risk factors in the Norwegian cohort were not measured during pregnancy, making it difficult to draw valid inference about the *in utero* influences.

### Possible mechanisms

Cardiovascular disease risk can be transmitted from mothers to children through multiple inter-related pathways involving genetic pre-disposition, intrauterine environment, and post-natal shared familial environment (e.g. diet, physical activity).^37–39^ Along the paternal line, disease risk transmission can occur through mechanisms that exclude the intrauterine environment. The paternal hazard estimates in this study likely reflect shared genetic and post-natal familial environmental factors. Consequently, the relationship between maternal CVD and offspring CVD risk may be partly due to shared genetic liability, as evidenced by the increased CVD risk in offspring with paternal CVD. A population-based cohort study in the UK^39^ revealed that maternal hypertensive disorders of pregnancy were associated with elevated offspring blood pressure in childhood, but not with other cardiovascular biomarkers, indicating the involvement of genetic factors shared between mothers and offspring. Genetic factors, however, do not typically act alone but interact with the environmental risk factors to contribute to the development of CVD.^38^

The stronger maternal–offspring associations in this study, as compared to paternal–offspring associations, suggest that genetic or shared familial factors alone do not fully explain the maternal transmission of CVD risk and may partially result from the mother’s unique *in utero* environment. Cardiovascular dysfunction in pregnancy can affect maternal cardiac output and placental perfusion, potentially leading to complications.^40^ A growing body of epidemiological research indicates that pre-existing maternal heart diseases negatively affect placental function, influencing the *in utero* environment and contributing to obstetric and neonatal complications.^41–43^ An intrauterine environment impaired by maternal CVD could compromise long-term cardiovascular health of offspring through epigenetic modifications of gene expression or suboptimal foetal growth and development.^4,44,45^

Further, the stronger maternal–offspring association for CVD may be linked to the use of CVD medications or withdrawal from such medications during pregnancy, which may negatively affect the foetus.^46^ The amplified maternal effects may also arguably arise from a greater maternal than paternal influence on child’s dietary and other behavioural patterns post-natally. However, previous investigations have reported concordance of lifestyle and other CVD risk factors between spouses.^24,25^ Another explanation could be greater genetic susceptibility attributable to mitochondrial DNA, which is inherited through the mother, although evidence is currently lacking to support this hypothesis.^47^

In our study, preterm birth and SGA showed little or no impact in the observed maternal–offspring CVD association. Our finding that the co-occurrence of mother’s CVD and psychiatric disease exacerbates offspring vulnerability to CVD suggests a possible role of the adverse *in utero* environment. Psychiatric disorders, including depression, are highly prevalent in patients with CVD, especially in women with CVD.^22,48^ This comorbidity might be associated with adverse medical prognosis and poor quality of life through complex mechanisms, such as harmful effects of antidepressant medications, dysfunction of the hypothalamic–pituitary–adrenal axis, non-adherence with cardiac treatment, and unhealthy behaviours such as poor diet, physical inactivity, and alcohol consumption.^48,49^ While preliminary evidence supports each of these mechanisms, there is limited knowledge about how they operate during pregnancy to impact offspring health in women with CVD and comorbid psychiatric illnesses. This remains an important area for future research.

### Policy implications

Given the rising prevalence of CVD and its risk factors among pregnant women worldwide,^2^ the study findings underscore the need for early identification and management of CVD in women of reproductive age. Pre-natal prevention strategies should emphasize lifestyle modifications, such as smoking cessation, healthy diet, physical activity, and controlling BMI, blood pressure, and cholesterol levels, as recommended by the European Society of Cardiology.^50,51^ Further research is crucial to understand the complex inter-play between maternal genetic and intrauterine risk factors in the aetiology and development of CVD in offspring.^38^

### Strengths and limitations

A major strength of the study is its large nationwide sample with long follow-up, which allowed us to analyse different types of parental and offspring cardiovascular conditions. There were no losses to follow-up in our study due to the high coverage and quality of the Swedish registers.^14,16–18^ To strengthen causal inference of the effect estimates, we were able to account for not only important maternal covariates but also unmeasured confounders, involving genetics and the shared family environment. Furthermore, to filter out offspring CVD diagnoses related to perinatal complications, we excluded all children diagnosed with CVD before reaching one year of age. We also excluded offspring with diagnosis of congenital malformations throughout the study period.

Nevertheless, our study has limitations. First, residual confounding may remain due to lack of data on maternal pre-pregnancy lifestyle factors, including diet, alcohol drinking, and physical activity. This particularly limits interpretation of the AFE, which is by definition a causal metric and relies on no-confounding assumption, which is hardly met in observational studies.^21^ The mediation analysis is additionally prone to bias due to unmeasured mediator–outcome confounding, which does not bias the exposure effect but can lead to invalid estimation of the mediated effect. Second, although we minimized the risk of false-positive CVD diagnoses by using minimum two ICD records in the outpatient register, misclassification may still occur. Maternal CVD is more likely to be diagnosed due to thorough screening during pregnancy, whereas mild paternal CVD may go undiagnosed. Further, healthcare seeking behaviour differences between mothers with and without CVD could lead to differential misclassification of offspring CVD. However, the high validity of cardiovascular diagnoses in the Swedish inpatient register^16^ suggests that any misclassification bias is unlikely to impact our conclusions. Third, the paternal CVD as negative control is only helpful for an indirect evaluation of the role of intrauterine environment. It would be more appropriate to use specific measures of placental dysfunction for identifying the mechanisms of interest. Fourth, the true association between maternal and offspring CVD may be underestimated due to the exclusion of foetal deaths and stillbirths if these are affected by the exposure, known as ‘live-birth bias’.^52^ Fifth, the follow-up of our study subjects only extends to young adulthood, limiting extrapolation of findings beyond this specific age range. Finally, despite using a large sample, the study was not well-powered to analyse finer categories of CVD. We acknowledge that the precise pathophysiological mechanisms underlying the observed associations are likely different for different types of maternal CVD and warrant attention in future research.

## Conclusions

This large cohort study shows that the offspring of mothers with pre-existing CVD have elevated risk of developing non-congenital CVD in childhood, adolescence, and young adulthood. The maternal influence on CVD risk was stronger than paternal influence, with genetic and familiar factors only partially accounting for this difference.

## Supplementary Material

ehae547_Supplementary_Data

## Data Availability

The data underlying this article cannot be shared publicly due to ethical reasons.
