# Supplementary material for: Pre-existing maternal cardiovascular disease and the risk of offspring cardiovascular disease from infancy to early adulthood
Source: Eur Heart J. 2024 Sep 4;45(38):4111–23. doi: 10.1093/eurheartj/ehae547 (PMC11458151; doi:10.1093/eurheartj/ehae547)
Supplement: ehae547_Supplementary_Data [file ehae547_supplementary_data.pdf]

Supplementary Online Content

Hossin et al. Pre-existing maternal and offspring cardiovascular disease into adulthood

Supplementary methods

Figure S1. Log-minus-log survival curves for pre-existing maternal cardiovascular disease (CVD) and CVD in offspring aged 1-29 years

Figure S2. Plots showing adjusted associations of maternal and paternal CVD with offspring CVD, stratified by parental age groups: singleton offspring live-born without major malformations in Sweden 1992-2019

Table S1. International Classification of Disease (ICD) codes for offspring morbidity

Table S2. International Classification of Disease (ICD) codes for maternal morbidity

Table S3. Frequency of major maternal cardiovascular disease subtypes by maternal age groups among children born to mothers with any pre-existing maternal cardiovascular disease (N=26 471)

Table S4. Frequency of major cardiovascular disease subtypes among children diagnosed with any cardiovascular disease (N=17 382)

Table S5. Incidence rates, rate differences and hazard ratios of offspring cardiovascular disease by the presence of pre-existing paternal cardiovascular disease: singletons live-born without major malformations in Sweden 1992-2019 (N=2 569 079)

Table S6. Incidence rates and hazard ratios of offspring CVD by preterm birth: singletons live-born without major malformations in Sweden (N=2 597 786)

Table S7. Mediation of the association between maternal pre-existing CVD and offspring CVD by preterm birth: singleton offspring live-born without major malformations in Sweden 1992-2019 (N=2 597 786)

Table S8. Incidence rates and hazard ratios of offspring cardiovascular disease by subtypes of pre-existing maternal and paternal cardiovascular disease: singleton offspring live-born without major malformations in Sweden 1992-2019

Table S9. Contribution of maternal cohabitation, smoking, and body mass index to the associations between maternal and offspring cardiovascular disease: singleton offspring live-born without major malformations in Sweden 1992-2019 (N=2 626 312)

Table S10. Incidence rates and hazard ratios of offspring cardiovascular disease by pre-existing maternal cardiovascular disease, excluding preeclampsia and gestational diabetes: singleton offspring live-born without major malformations in Sweden 1992-2019 (N=2 499 321)

Table S11. Incidence rates and hazard ratios of offspring CVD by duration of pre-existing maternal cardiovascular disease: singletons live-born without major malformations in Sweden (N=2 597 786)

Table S12. Sensitivity analysis of the parental-offspring cardiovascular disease associations, restricted to the birth cohorts 2001-2019: singletons live-born without major malformations in Sweden

Table S13. Sensitivity analysis of the maternal-offspring cardiovascular disease associations, restricted to children of mothers born in Sweden: singletons live-born without major malformations in Sweden (N=2 059 772)

Table S14. Sensitivity analysis of the maternal-offspring cardiovascular disease associations, with additional inclusion of maternal cardiovascular diagnoses from the Medical Birth Register: singletons live-born without major malformations in Sweden (N=2 597 786)

Supplementary references

Supplementary methods

Description of the Swedish registers used in the study:

The Swedish Medical Birth Register

The Swedish Medical Birth Register (MBR) retrieves data on prenatal, obstetric, and neonatal characteristics, including maternal diagnoses, based on medical records from the prenatal care, the delivery care, and the neonatal care. Founded in 1973, the MBR covers >98% of all births occurring in Sweden[1,2].

The Swedish National Patient Register

The National Patient Register (NPR) consists of the inpatient and outpatient registers and holds data on health care from both public and private caregivers. The inpatient register, also known as hospital discharge register, was initiated in 1964 (psychiatric diagnoses since 1973), but complete nationwide coverage was obtained in 1987. The outpatient register was launched in 2001 and records information on visits to specialized outpatient care. The diagnoses of diseases recorded in the NPR are identified using the Swedish version of the International Classification of Disease (ICD) codes[3].

The Swedish Cause of Death Register

The Swedish Cause of Death Register (CDR) is an excellent source of data for register-based research and contains information on all deaths in Sweden since 1952. The register is known for its high quality and complete data and is linked to other national registers through unique personal identification numbers[4]. Unlike the NPR that uses the Swedish version of ICD codes to classify diseases, the CDR uses the international version of the classification system to facilitate international comparison of cause-specific mortality statistics[5].

The Swedish Total Population Register

Since 1968, the Total Population Register (TPR) contains sociodemographic information (eg, sex, country of birth) as well as data on important life events including dates of birth, death and migration of all residents in Sweden. The TPR allows complete follow up and censoring of individuals and thereby minimizes the risk of selection bias[6].

The Swedish Education Register

Since 1985, the Education Register in Sweden contains annually updated data on completed education, collected from more than 30 different sources including the schools and education providers and questionnaire surveys. Data on education is coded according to the Swedish education nomenclature SUN, a classification system which was later adapted to the International Standard Classification of Education (ISCED 97) in 2000, distinguishing between the level and type of education[7]

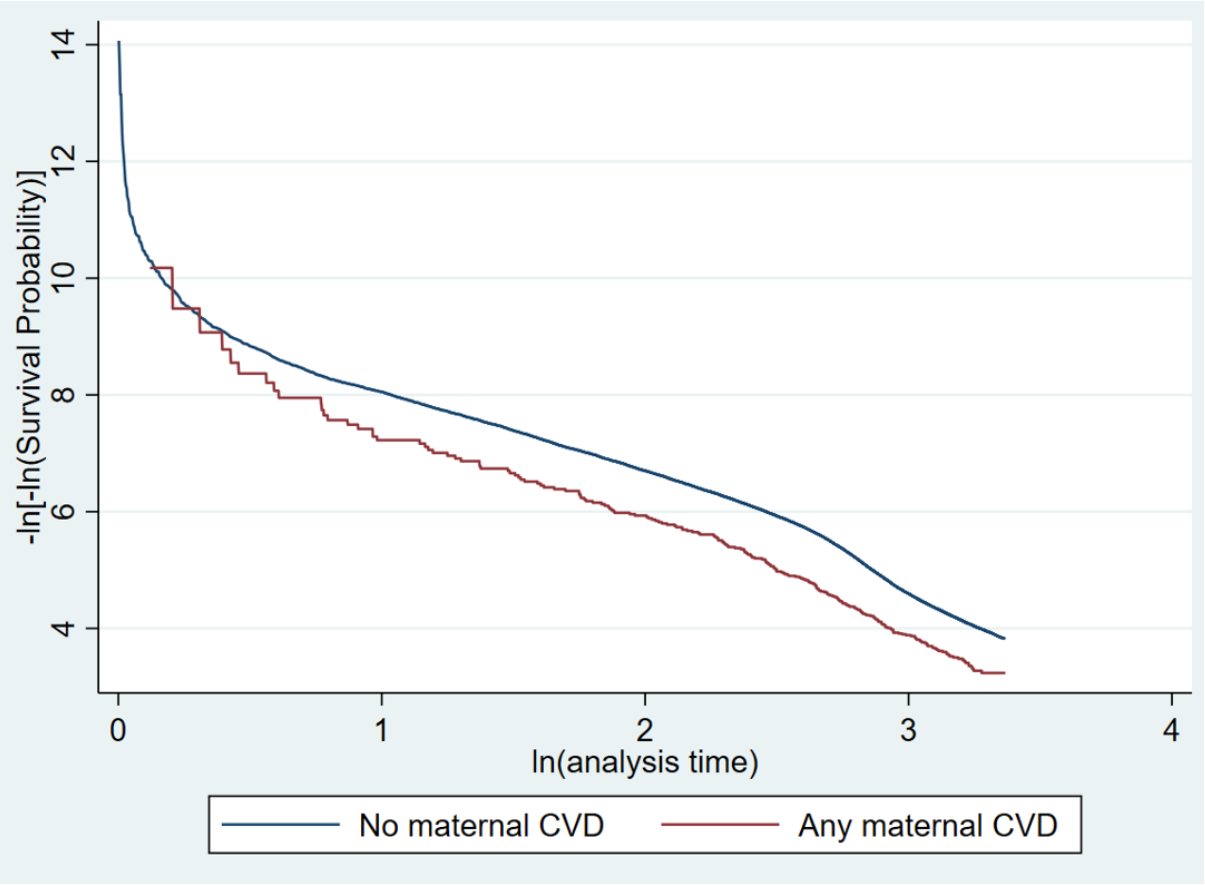

Figure S1. Log-minus-log survival curves for pre-existing maternal cardiovascular disease (CVD) and CVD in offspring aged 1-29 years

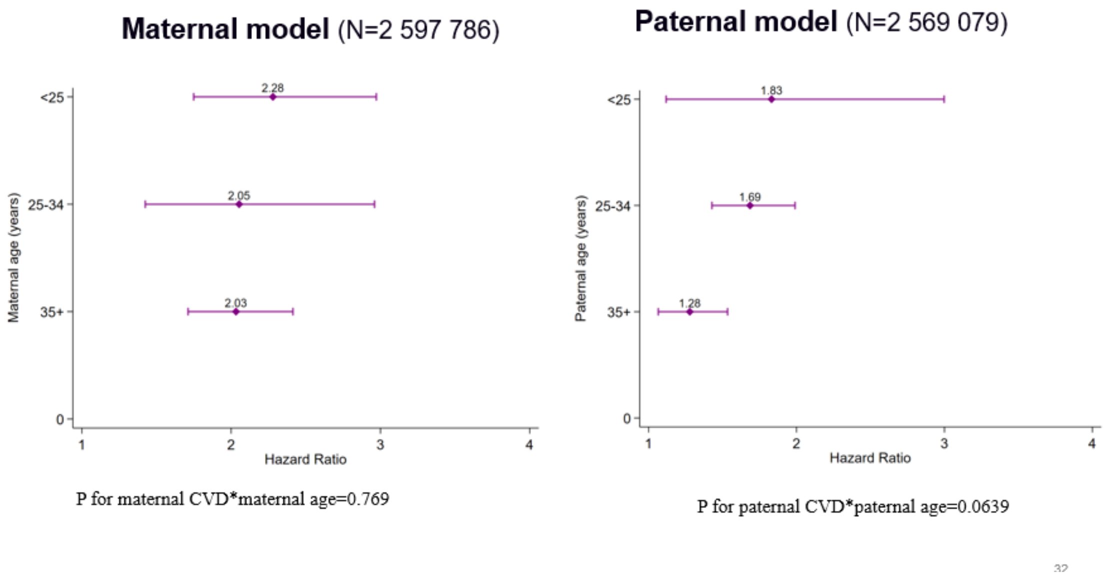

Note: CVD, Cardiovascular disease. The hazard ratios were adjusted for offspring sex and birth year and mother's age at delivery, parity, country of birth, level of education, pre-gestational diabetes, parents' chronic kidney disease, parental history of any psychiatric disorders. The maternal and paternal CVD were mutually adjusted for each other. The paternal models were additionally adjusted for paternal age at childbirth.

Figure S2. Plots showing adjusted associations of maternal and paternal CVD with offspring CVD, stratified by parental age groups: singleton offspring live-born without major malformations in Sweden 1992-2019

Table S1. International Classification of Disease (ICD) codes for offspring morbidity

	**ICD-9** (1987-1996)	**ICD-10** (1997-2020)
Any CVD	390-459	I00-I99
CVD subtypes		
Ischaemic heart disease	410-414	I20-I25
Cerebrovascular disease	430-438	I60-I69, G45
Hypertensive disease	401-405	I10-I15
Arrhythmia	426-427	I44-I49
Congenital heart disease	745-747	Q20-Q26
Chronic kidney disease	585	N18
Offspring's major malformations	744B, 744E, 746X, 747F, 750A, 750B, 752F, 754D, 754G, 755G, 755A, 755B, 756B, 756X, 757D, 757X.	Q7550, Q175, Q180, Q181, Q249, Q250, Q270, Q289, Q314, Q315, Q320, Q381, Q523, Q53, Q65, Q665, Q666, Q667, Q668, Q669, Q690, Q699, Q703, Q760, Q799, Q825, Q829

Note: Diseases were defined using the Swedish version of the International Classification of Disease codes.

Table S2. International Classification of Disease (ICD) codes for maternal morbidity

	**ICD-9** (1987-1996)	**ICD-10** (1997-2020)
Any CVD	390-459	I00-I99
CVD subtypes		
Ischaemic heart disease	410-414	I20-I25
Cerebrovascular disease	430-438	I60-I69, G45
Hypertensive disease	401-405	I10-I15
Arrhythmia	426-427	I44-I49
Cardiomyopathy	425	I42, I43
Heart failure	428	I50
Valvular heart disease	394-397, 424	I05-I09, I34-I37
Congenital heart disease	745-747	Q20-Q26
Any psychiatric disorders	290-319	F00-F99
Pre-gestational diabetes	250, 648A	E10-E14, O24.1-O24.3
Chronic kidney disease	585	N18
Gestational diabetes	648W	O244
Pre-eclampsia	642E-642G	O14-15

Note: Diseases were defined using the Swedish version of the International Classification of Disease codes.

Table S3. Frequency of major maternal cardiovascular disease subtypes by maternal age groups among children born to mothers with any pre-existing maternal cardiovascular disease (N=26 471)

Pre-existing maternal CVD subtypes	Total (N= 26 471)	Maternal age groups		
		<25 yrs (N=2529)	25-34 yrs (N=16 300)	>34 yrs (N=7642)
	% (n)	% (n)	% (n)	% (n)
Ischemic heart disease	1.4 (378)	0.8 (21)	1.2 (190)	2.2 (167)
Cerebrovascular disease	9.9 (2632)	8.7 (219)	9.6 (1571)	11.0 (842)
Hypertensive disease	16.6 (4383)	11.1 (281)	14.4 (2351)	21.9 (1751)
Arrhythmia	35.5 (9395)	38.2 (967)	36.5 (5948)	32.5 (2480)
Valvular heart disease	7.0 (1839)	7.0 (176)	7.2 (1182)	6.3 (481)
Cardiomyopathy	1.3 (349)	1.7 (44)	1.2 (201)	1.4 (104)
Heart failure	1.7 (463)	2.7 (69)	1.8 (290)	1.4 (104)

Table S4. Frequency of major cardiovascular disease subtypes among children diagnosed with any cardiovascular disease (N=17 382)

**Offspring CVD subtypes**	n	**%**
Ischemic heart disease	70	0.4
Cerebrovascular disease	1350	7.8
Hypertensive disease	2118	12.2
Arrhythmia	6630	38.1
Cardiomyopathy	478	2.8
Heart failure	312	1.8
Valvular heart disease	351	2.0

Table S5. Incidence rates, rate differences and hazard ratios of offspring cardiovascular disease by the presence of pre-existing paternal cardiovascular disease: singletons live-born without major malformations in Sweden 1992-2019 (N=2 569 079)

	**Outcome: Any CVD in offspring**				
**Pre-existing paternal CVD**	No. of events	Rates[1]	Rate Difference (95% CI) [1]	Model 1 HR (95% CI)	Model 2 HR (95% CI)[2]
Any CVD					
No	16 985	50.3	0.0 (Ref.)	1.00 (Ref.)	1.00 (Ref.)
Yes	277	61.1	10.8 (3.6, 18.2)	1.50 (1.34, 1.70)	1.49 (1.32, 1.68)
**Pre-existing paternal CVD sub-groups**					
Vascular heart disease					
No	17 184	55.4	0.0 (Ref.)	1.00 (Ref.)	1.00 (Ref.)
Yes	78	53.4	-3.0 (-0.8.9, 14.8)	1.33 (1.06, 1.66)	1.25 (1.00, 1.57)
Arrhythmia					
No	17 165	50.4	0.0 (Ref.)	1.00 (Ref.)	1.00 (Ref.)
Yes	97	67.6	17.2 (3.7, 30.7)	1.72 (1.41, 2.10)	1.61 (1.31, 1.97)
Structural heart disease					
No	17 216	50.4	0.0 (Ref.)	1.00 (Ref.)	1.00 (Ref.)
Yes	46	65.8	15.4 (-3.7, 34.4)	1.78 (1.33, 2.37)	1.53 (1.14, 2.06)

	**Outcome: Arrhythmia in offspring[3]**				
**Pre-existing paternal CVD**					
Any CVD					
No	8184	22.5	0.0 (Ref.)	1.00 (Ref.)	1.00 (Ref.)
Yes	175	35.0	12.5 (7.3, 17.7)	1.72 (1.48, 2.00)	1.70 (1.46, 1.98)
**Pre-existing paternal CVD sub-groups**					
Vascular heart disease					
No	8328	22.7	0.0 (Ref.)	1.00 (Ref.)	1.00 (Ref.)
Yes	31	19.2	-3.5 (-10.3, 3.3)	0.94 (0.66, 1.34)	0.85 (0.59, 1.21)
Arrhythmia					
No	8259	22.5	0.0 (Ref.)	1.00 (Ref.)	1.00 (Ref.)
Yes	100	63.1	40.6 (28.2, 53.0)	3.16 (2.59, 3.85)	3.09 (2.52, 3.77)
Structural heart disease					
No	8337	22.7	0.0 (Ref.)	1.00 (Ref.)	1.00 (Ref.)
Yes	22	28.1	5.4 (-6.3, 17.2)	1.42 (0.93, 2.16)	1.03 (0.67, 1.58)

	**Outcome: Vascular disease in offspring**				
**Pre-existing Paternal CVD**					
Any CVD					
No	3550	1.0	0.0 (Ref.)	1.00 (Ref.)	1.00 (Ref.)
Yes	49	1.1	4.3 (-0.4, 9.0)	1.25 (0.94, 1.66)	1.18 (0.89, 1.57)
**Pre-existing maternal CVD sub-groups**					
Vascular heart disease					
No	3573	10.5	0.0 (Ref.)	1.00 (Ref.)	1.00 (Ref.)
Yes	26	17.8	7.3 (0.5, 14.1)	2.08 (1.42, 3.07)	1.93 (1.30, 2.87)

Arrhythmia					
No	3589	10.5	0.0 (Ref.)	1.00 (Ref.)	1.00 (Ref.)
Yes	10	6.9	-3.6 (-7.9, 0.8)	0.83 (0.45, 1.54)	0.72 (0.38, 1.35)
Structural heart disease					
No	3589	10.5	0.0 (Ref.)	1.00 (Ref.)	1.00 (Ref.)
Yes	10	14.2	3.8 (-5.1, 12.6)	1.79 (0.96, 3.33)	1.51 (0.80, 2.85)

Note: CI, confidence interval; CVD, cardiovascular disease; HR, hazard ratio.
[1]The crude incidence rates and rate differences were calculated per 100,000 person-years.
Model 1 shows the unadjusted HRs. **Model 2** was adjusted for offspring sex and birth year and mother's age at delivery, parity, country of birth, level of education, pre-gestational diabetes, parents' chronic kidney disease, parental history of any psychiatric disorders, maternal CVD, and paternal age at delivery
[2]The subgroups of paternal CVD were mutually adjusted for each other in model 2.
[3]Follow-up for offspring arrhythmia started from birth, instead of from first year of life (N=2 581 603).

Table S6. Incidence rates and hazard ratios of offspring CVD by preterm birth: singletons live-born without major malformations in Sweden (N=2 597 786)

	Any CVD in offspring			
**Preterm birth**	No. of events	Rates[1]	Model 1 HR (95% CI)	Model 2 HR (95% CI)
No	16 429	50.0	1.00 (Ref.)	1.00 (Ref.)
Yes	953	61.1	1.21 (1.13, 1.29)	1.19 (1.12, 1.27)

Note: CI, confidence interval; CVD, cardiovascular disease; HR, hazard ratio.
[1]Crude incidence rates per 100,000 child-years.
Model 1 shows the unadjusted HRs. **Model 2** was adjusted for offspring sex and birth year and mother's age at delivery, parity, country of birth, level of education, pre-gestational diabetes, parents' chronic kidney disease, parental history of any psychiatric disorders as well as maternal CVD.

Table S7. Mediation of the association between maternal pre-existing CVD and offspring CVD by preterm birth: singleton offspring live-born without major malformations in Sweden 1992-2019 (N=2 597 786)

**Mediation parameters**	IRR (95% CI)
Total effect	2.07 (1.81, 2.37)
Natural direct effect	2.02 (1.76, 2.31)
Natural indirect effect	1.03 (1.01, 1.05)
Proportion mediated	5%

Note: CI, confidence interval; CVD, cardiovascular disease; IRR, Incidence Rate Ratio.
The estimates were obtained from a Generalized Poisson Linear Model and were adjusted for offspring sex and birth year and mother's age at delivery, parity, country of birth, level of education, pre-gestational diabetes, parents' chronic kidney disease, parental history of any psychiatric disorders as well as paternal CVD.

Table S8. Incidence rates and hazard ratios of offspring cardiovascular disease by subtypes of pre-existing maternal and paternal cardiovascular disease: singleton offspring live-born without major malformations in Sweden 1992-2019

	**Maternal models** (N=2 597 786)			
**Pre-existing maternal CVD subtypes**	No. of events	Rates[1]	Model 1 HR (95% CI)	Model 2[2] HR (95% CI)
Ischemic heart disease				
No	17 377	50.5	1.00 (Ref.)	1.00 (Ref.)
Yes	<6*	112.7	2.50 (1.04, 6.00)	2.47 (1.03, 5.94)
Cerebrovascular disease				
No	17 369	50.5	1.00 (Ref.)	1.00 (Ref.)
Yes	13	41.6	0.97 (0.57, 1.68)	0.94 (0.55, 1.62)
Hypertensive disease				
No	17 348	50.4	1.00 (Ref.)	1.00 (Ref.)
Yes	34	86.7	2.36 (1.68, 3.31)	2.31 (1.64, 3.25)
Arrhythmia				
No	17 280	50.3	1.00 (Ref.)	1.00 (Ref.)
Yes	102	117.9	3.14 (2.58, 3.82)	3.06 (2.51, 3.71)
Cardiomyopathy/heart failure				
No	17 371	50.4	1.00 (Ref.)	1.00 (Ref.)
Yes	11	163.6	4.62 (2.56, 8.35)	4.53 (2.50, 8.18)
Valvular heart disease				
No	17 369	50.5	1.00 (Ref.)	1.00 (Ref.)
Yes	13	86.8	2.22 (1.29, 3.83)	2.20 (1.27, 3.78)
Congenital heart disease				
No	17 360	50.5	1.00 (Ref.)	1.00 (Ref.)
Yes	22	52.0	1.42 (0.94, 2.16)	1.37 (0.90, 2.09)
	**Paternal models** (N=2 569 079)			
**Pre-existing paternal CVD subtypes**				
Ischemic heart disease				
No	17 232	50.4	1.00 (Ref.)	1.00 (Ref.)
Yes	30	73.1	1.59 (1.11, 2.28)	1.62 (1.13, 2.32)
Cerebrovascular disease				
No	17 244	50.5	1.00 (Ref.)	1.00 (Ref.)
Yes	18	43.7	1.06 (0.67, 1.68)	1.05 (0.66, 1.66)
Hypertensive disease				
No	17 228	50.5	1.00 (Ref.)	1.00 (Ref.)
Yes	34	45.8	1.27 (0.91, 1.78)	1.27 (0.90, 1.79)
Arrhythmia				
No	17 165	50.4	1.00 (Ref.)	1.00 (Ref.)
Yes	97	67.6	1.72 (1.41, 2.10)	1.70 (1.39, 2.07)
Cardiomyopathy/heart failure				
No	17 245	50.4	1.00 (Ref.)	1.00 (Ref.)
Yes	17	100.5	2.70 (1.68, 4.34)	2.67 (1.66, 4.30)
Valvular heart disease				
No	17 246	50.5	1.00 (Ref.)	1.00 (Ref.)
Yes	16	60.9	1.61 (0.98, 2.62)	1.60 (0.98, 2.62)
Congenital heart disease				
No	17 241	50.5	1.00 (Ref.)	1.00 (Ref.)
Yes	21	56.6	1.62 (1.05, 2.48)	1.57 (1.02, 2.41)

Note: CI, confidence interval; CVD, cardiovascular disease; HR, hazard ratio.
[1]Crude incidence rates per 100,000 person-years.
Model 1 shows the unadjusted HRs. **Model 2** was adjusted for offspring sex and birth year and mother's age at delivery, parity, country of birth, level of education, pre-gestational diabetes, parents' chronic kidney disease, and parental history of any psychiatric disorders. The maternal and paternal CVD subtypes were mutually adjusted for each other.
[2]The paternal model 2 was additionally adjusted for paternal age at delivery.
*Exact count is suppressed for confidentiality reason.

Table S9. Contribution of maternal cohabitation, smoking, and body mass index to the associations between maternal and offspring cardiovascular disease: singleton offspring live-born without major malformations in Sweden 1992-2019 (N=2 626 312)*

**Pre-existing maternal CVD**	**Any CVD in offspring**				
	No. of events	Rates[1]	Model 1 HR (95% CI)	Model 2 HR (95% CI)	Model 3 HR (95% CI)
Any CVD					
No	17 293	50.2	1.00 (Ref.)	1.00 (Ref.)	1.00 (Ref.)
Yes	219	85.2	2.16 (1.89-2.47)	2.11 (1.84, 2.41)	2.09 (1.83, 2.39)
**Pre-existing maternal CVD subgroups**[2]					
Vascular disease					
No	17 460	50.4	1.00 (Ref.)	1.00 (Ref.)	1.00 (Ref.)
Yes	52	70.5	1.78 (1.35, 2.33)	1.62 (1.23, 2.13)	1.60 (1.22, 2.11)
Arrhythmia					
No	17 409	50.3	1.00 (Ref.)	1.00 (Ref.)	1.00 (Ref.)
Yes	103	118.8	3.16 (2.61, 3.84)	2.95 (2.42, 3.59)	2.95 (2.43, 3.59)
Structural disease					
No	17 471	50.4	1.00 (Ref.)	1.00 (Ref.)	1.00 (Ref.)
Yes	41	73.2	1.97 (1.45, 2.68)	1.57 (1.15, 2.14)	1.57 (1.15, 2.14)

Note: CI, confidence interval; CVD, cardiovascular disease; HR, hazard ratio.
*Multiple imputation analysis was performed using 15 imputed datasets
[1]Incidence rates per 100,000 person-years.
Model 1 shows the unadjusted HRs. **Model 2** was adjusted for offspring sex and birth year and mother's age at delivery, parity, country of birth, level of education, pre-gestational diabetes, parents' chronic kidney disease, parental history of any psychiatric disorders as well as paternal CVD. The subgroups of maternal CVD were mutually adjusted for each other in the adjusted models. **Model 3** was additionally adjusted for maternal cohabitation, smoking, and body mass index.

Table S10. Incidence rates and hazard ratios of offspring cardiovascular disease by pre-existing maternal cardiovascular disease, excluding preeclampsia and gestational diabetes: singleton offspring live-born without major malformations in Sweden 1992-2019 (N=2 499 321)

**Pre-existing maternal CVD**	No. of events	Rates[1]	Model 1 HR (95% CI)	Model 2 HR (95% CI)
Any CVD				
No	16 493	50.0	1.00 (Ref.)	1.00 (Ref.)
Yes	209	86.1	2.19 (1.91, 2.51)	2.14 (1.86, 2.45)
**Pre-existing maternal CVD sub-groups**				
Vascular heart disease				
No	16 653	50.3	1.00 (Ref.)	1.00 (Ref.)
Yes	49	72.0	1.82 (1.37, 2.40)	1.65 (1.24, 2.19)
Arrhythmia				
No	16 603	50.1	1.00 (Ref.)	1.00 (Ref.)
Yes	99	119.3	3.19 (2.62, 3.89)	2.98 (2.44, 3.64)
Structural heart disease				
No	16 663	50.3	1.00 (Ref.)	1.00 (Ref.)
Yes	39	70.4	1.89 (1.38, 2.59)	1.49 (1.08, 2.05)

Note: CI, confidence interval; CVD, cardiovascular disease; HR, hazard ratio.
[1]Crude incidence rates per 100,000 person-years.
Model 1 shows the unadjusted HRs. **Model 2** was adjusted for offspring sex and birth year and mother's age at delivery, parity, country of birth, level of education, pre-gestational diabetes, parents' chronic kidney disease, parental history of any psychiatric disorders as well as paternal CVD. The subgroups of maternal CVD were mutually adjusted for each other in model 2.

Table S11. Incidence rates and hazard ratios of offspring CVD by duration of pre-existing maternal cardiovascular disease: singletons live-born without major malformations in Sweden (N=2 597 786)

**Duration of pre-existing maternal CVD**	**Any CVD in offspring**			
	No. of events	Rates[1]	Model 1 HR (95% CI)	Model 2 HR (95% CI)
No CVD	17 165	50.2	1.00 (Ref.)	1.00 (Ref.)
CVD <2 years before conception	93	100.0	2.50 (2.03, 3.06)	2.46 (2.00, 3.01)
CVD 2–5 years before conception	61	80.9	1.88 (1.46, 2.42)	1.84 (1.43, 2.37)
CVD >5 years before conception	63	71.5	2.00 (1.56, 2.56)	1.93 (1.51, 2.48)

Note: CI, confidence interval; CVD, cardiovascular disease; HR, hazard ratio.
[1]Crude incidence rates per 100,000 child-years.
Model 1 shows the unadjusted HRs. **Model 2** was adjusted for offspring sex and birth year and mother's age at delivery, parity, country of birth, level of education, pre-gestational diabetes, parents' chronic kidney disease, parental history of any psychiatric disorders as well as paternal CVD.
The Wald test suggested that the HR 2.46 was not statistically different from the HR 1.84 (p-for-difference 0.081) and the HR 1.93 (p-for-difference 0.141)

Table S12. Sensitivity analysis of the parental-offspring cardiovascular disease associations, restricted to the birth cohorts 2001-2019: singletons live-born without major malformations in Sweden

	**Any CVD in offspring**			
	No. of events	Rates[1]	Model 1 HR (95% CI)	Model 2 HR (95% CI)[2]
**Any pre-existing maternal CVD** (N=1 801 738)				
No	4940	31.0	1.00 (Ref.)	1.00 (Ref.)
Yes	109	61.3	2.13 (1.76, 2.57)	2.12 (1.75, 2.56)
**Any pre-existing paternal CVD** (N=1 777 790)				
No	4877	31.1	1.00 (Ref.)	1.00 (Ref.)
Yes	126	41.6	1.43 (1.20, 1.71)	1.44 (1.20, 1.72)

Note: CI, confidence interval; CVD, cardiovascular disease; HR, hazard ratio.
[1]Crude incidence rates per 100,000 person-years.
Model 1 shows the unadjusted HRs. **Model 2** was adjusted for offspring sex and birth year and mother's age at delivery, parity, country of birth, level of education, pre-gestational diabetes, parents' chronic kidney disease, and parental history of any psychiatric disorders. Model 2 was also mutually adjusted for maternal and paternal CVD.
[2]The paternal model 2 was additionally adjusted for paternal age at delivery.

Table S13. Sensitivity analysis of the maternal-offspring cardiovascular disease associations, restricted to children of mothers born in Sweden: singletons live-born without major malformations in Sweden (N=2 059 772)

	**Any CVD in offspring**			
	No. of events	Rates[1]	Model 1 HR (95% CI)	Model 2 HR (95% CI)
**Any pre-existing maternal CVD**				
No	14 753	52.0	1.00 (Ref.)	1.00 (Ref.)
Yes	194	85.9	2.13 (1.85, 2.46)	2.10 (1.82, 2.42)

Note: CI, confidence interval; CVD, cardiovascular disease; HR, hazard ratio.
[1]Crude incidence rates per 100,000 person-years.
Model 1 shows the unadjusted HRs. **Model 2** was adjusted for offspring sex and birth year and mother's age at delivery, parity, country of birth, level of education, pre-gestational diabetes, parents' chronic kidney disease, parental history of any psychiatric disorders as well as paternal CVD.

Table S14. Sensitivity analysis of the maternal-offspring cardiovascular disease associations, with additional inclusion of maternal cardiovascular diagnoses from the Medical Birth Register: singletons live-born without major malformations in Sweden (N=2 597 786)

	Any CVD in offspring			
	No. of events	Rates[1]	Model 1 HR (95% CI)	Model 2 HR (95% CI)
**Any pre-existing maternal CVD**				
No	17 151	50.2	1.00 (Ref.)	1.00 (Ref.)
Yes	231	86.4	2.14 (1.88, 2.43)	2.09 (1.84, 2.38)

Note: CI, confidence interval; CVD, cardiovascular disease; HR, hazard ratio.
[1]Crude incidence rates per 100,000 person-years.
Model 1 shows the unadjusted HRs. **Model 2** was adjusted for offspring sex and birth year and mother's age at delivery, parity, country of birth, level of education, pre-gestational diabetes, parents' chronic kidney disease, parental history of any psychiatric disorders as well as paternal CVD.

Supplementary references

1. Swedish National Board of Health and Welfare. *Det Statistiska Registrets Framställning Och Kvalitet: Medicinska Födelseregistret (Production and Quality of the statistical Register: Medical Birth Register*.; 2021. Accessed December 28, 2021. https://www.socialstyrelsen.se/globalassets/sharepoint-dokument/artikelkatalog/statistik/2021-9-7547.pdf

2. Cnattingius S, Källén K, Sandström A, et al. The Swedish medical birth register during five decades: documentation of the content and quality of the register. *Eur J Epidemiol*. Published online 2023:1-12. doi:10.1007/s10654-022-00947-5

3. Ludvigsson JF, Andersson E, Ekbom A, et al. External review and validation of the Swedish national inpatient register. *BMC Public Health*. 2011;11. doi:10.1186/1471-2458-11-450

4. Ludvigsson JF, Otterblad-Olausson P, Pettersson BU, Ekbom A. The Swedish personal identity number: Possibilities and pitfalls in healthcare and medical research. *Eur J Epidemiol*. 2009;24(11):659-667. doi:10.1007/s10654-009-9350-y

5. Brooke HL, Talbäck M, Hörnblad J, et al. The Swedish cause of death register. *Eur J Epidemiol*. 2017;32(9):765-773. doi:10.1007/s10654-017-0316-1

6. Ludvigsson JF, Almqvist C, Bonamy AKE, et al. Registers of the Swedish total population and their use in medical research. *Eur J Epidemiol*. 2016;31(2):125-136. doi:10.1007/s10654-016-0117-y

7. Ludvigsson JF, Svedberg P, Olén O, Bruze G, Neovius M. The longitudinal integrated database for health insurance and labour market studies (LISA) and its use in medical research. *Eur J Epidemiol*. 2019;34(4):423-437. doi:10.1007/s10654-019-00511-8
